# Facial and Oral Manifestations Following COVID-19 Vaccination: A Survey-Based Study and a First Perspective

**DOI:** 10.3390/ijerph18094965

**Published:** 2021-05-07

**Authors:** Marta Mazur, Irena Duś-Ilnicka, Maciej Jedliński, Artnora Ndokaj, Joanna Janiszewska-Olszowska, Roman Ardan, Malgorzata Radwan-Oczko, Fabrizio Guerra, Valeria Luzzi, Iole Vozza, Roberto Marasca, Livia Ottolenghi, Antonella Polimeni

**Affiliations:** 1 Department of Oral and Maxillofacial Sciences, Sapienza University of Rome, Via Caserta 6, 00161 Rome, Italy; maciej.jedlinski@pum.edu.pl (M.J.); artnora.ndokaj@uniroma1.it (A.N.); fabrizio.guerra@uniroma1.it (F.G.); valeria.luzzi@uniroma1.it (V.L.); iole.vozza@uniroma1.it (I.V.); livia.ottolenghi@uniroma1.it (L.O.); antonella.polimeni@uniroma1.it (A.P.); 2 Department of Oral Pathology, Wroclaw Medical University, ul. Krakowska 26, 52-425 Wrocław, Poland; irena.dus-ilnicka@umed.wroc.pl (I.D.-I.); malgorzata.radwan-oczko@umed.wroc.pl (M.R.-O.); 3 Department of Interdisciplinary Dentistry, Pomeranian Medical University in Szczecin, 70-111 Szczecin, Poland; jjo@pum.edu.pl; 4 Department of Economic Sciences, Koszalin University of Technology, 75-343 Koszalin, Poland; roman.ardan@tu.koszalin.pl; 5 Paediatric Dentistry Unit, Head and Neck Integrated Department, AOU Policlinico Umberto I of Rome, Viale Regina Elena 287/b, 00161 Rome, Italy; r.marasca@policlinicoumbero1.it

**Keywords:** COVID-19, COVID-19 vaccines, vaccines, BNT162b2, mRNA-1273, survey, questionnaire, oral signs, facial signs, orofacial manifestations

## Abstract

(1) Background: Severe acute respiratory syndrome Coronavirus-2 (SARS-CoV-2) emerged in Wuhan, China, in late 2019. The development of effective and safe vaccines against SARS-CoV-2 has been extremely fast. The list of orofacial adverse effects of BNT162b2 and mRNA-1273 vaccines based on the clinical trials are reported to be rare. The aim of this study was to investigate the facial and oral manifestations of COVID-19 vaccination using a survey-based study. (2) Methods: The questionnaire was developed using Google Forms and sent anonymously to a total of 700 subjects (medical personnel) in Poland, Italy, and other EU countries. (3) Results: 223 people answered the questionnaire, mainly vaccinated with BNT162b2. Only 3.1% and 5.4% experienced oral and facial symptoms, respectively. General diseases presence and age have significant influence on the probability of oral symptoms occurrence after the second dose. Facial symptoms are correlated with general disease; autoimmune pathologies and age, at first and second dose, respectively. Gender, smoking and regular medication intake have significant influence on the probability of taking an absence day. Gender, age, and smoking have a significant influence on the duration of symptoms after second dose. (4) Conclusions: Based on the results of this preliminary survey, there is no observed significant correlation between vaccine administration for COVID-19 and facial and oral manifestations.

## 1. Introduction

Severe acute respiratory syndrome coronavirus-2 (SARS-CoV-2) emerged in Wuhan, China, in late 2019. The COVID-19 pandemic quickly spread globally and has since afflicted tens of millions of people worldwide [1]. There have been conflicting reports in the literature of the prevalence of various oral signs and symptoms in patients with COVID-19. Among these, gustatory impairment was the most common [2,3]. A multitude of clinical conditions affecting the oral cavity have been reported, including white and erythematous lesions, ulceration, blisters, petechiae, vascular disorders, stomatitis, and necrotizing periodontal disease. The majority of these clinical symptoms were presumably the aftermath of COVID-19 and treatment-related immunosuppression and predisposition to opportunistic infections such as candidiasis. However, some authors underline direct infection by SARS-CoV-2 and its replication in oral keratinocytes and fibroblasts in minor salivary glands, causing oral ulceration, necrosis, and hemorrhage [2,3]. 

The development of effective and safe vaccines against SARS-CoV-2 has been extremely fast [4]. The development of BNT162b2 started in early January, 2020, after the release of the SARS-CoV-2 genetic sequence by the Chinese Center for Disease Control and Prevention, and its global dissemination by the Global Initiative on Sharing All Influenza Data (GISAID). Initial safety and efficacy for BNT162b2 (Pfizer and BioNtech) has been demonstrated in a study on a total of 43,584 participants randomized to vaccine and placebo [5]. Product monographs including information from the trial are available. A study by Cirillo [6] documented the list of orofacial adverse effects (OAEs) of BNT162b2 and mRNA-1273 available to patients and healthcare providers. The listed OAEs differ between countries and regions (USA, Canada, EU, UK) and between vaccines provided to patients and healthcare providers. The reported OAEs are (i) swelling of the lips, face, and/or throat, and (ii) temporary unilateral facial drooping (Bell’s palsy) and are rare, occurring in up to 1 in 1000 people [6].

As vaccination campaigns are rolled out worldwide, and with several billion doses predicted to be administered in the near future [7], dentists among other clinical specialists are expected to provide treatment to the vaccinated population, and it is important that Hill’s criteria of causal inference are adhered to and rigorous anamnestic records are taken in order to deliver the best care to patients [8]. In Europe, the vaccination campaign policies are organized at local and regional level, and each member state has autonomy in determining which population groups to be vaccinated first. For example, in Italy and Poland, priority was given to medical personnel, while in Germany and the Netherlands, the older part of the population. 

Therefore, the aim of this study was to investigate the facial and oral manifestations of COVID-19 vaccination using a survey-based study. 

## 2. Materials and Methods

### 2.1. Population

*Inclusion criteria*

- medical working staff (nurses, medical doctors, dentists);
- have access to COVID-19 vaccines;
- has been vaccinated with at least one dose.

The study was approved by the Institutional Review Board of territorial NHS facilities (n. 080420). The survey was carried out during the first 2 weeks of March 2021, approximately 2 months after the second dose for all the participants.

The questionnaire was developed using Google Forms and sent anonymously to a total of 700 subjects (medical personnel) in Poland, Italy, and other EU countries.

The questionnaire consisted of four parts: Part A—geographic, demographic and professional data (questions n = 5); Part B—COVID-19 vaccine, number of doses, date of inoculation, premedication, general symptoms (n = 11); Part C—facial and oral manifestations (n = 4); and Part D—underlying medical conditions and lifestyle factors (n = 9).

The facial and oral manifestation evaluated in this study were: (i) changes in sensitivity, facial paresis and facial aesthetic paresis; (ii) burning sensations, oral aphtous-like lesions, taste alteration, xerostomia, tongue depapillation, pain, stomatitis/mucositis, commissural cheilitis, oral candidiasis, respectively.

The queries related to Parts A and D are reported in Table 1, and to Parts B and C in Table 2. 

### 2.2. Statistical Analysis

Correlation coefficients between age, gender and symptoms-related characteristics were calculated. Logistic regression models for the probability of the occurrence of oral and facial symptoms and of taking an absence day were examined. Linear regression models for the duration of symptoms were also explored. The influence of demographic and health factors such as gender, age, presence of autoimmune pathology, diabetes and/or general diseases, being a smoker and/or allergic, and regular medication intake were studied. Optimal models were chosen by Akaike information criterion (AIC). The results were considered statistically significant at *p* < 0.1. The R statistical program (The R Foundation for Statistical Computing, Wirtschaftsuniversität Wien, Vienna, Austria) was used for the calculations. 

## 3. Results

### 3.1. Data

**Figure 1:**
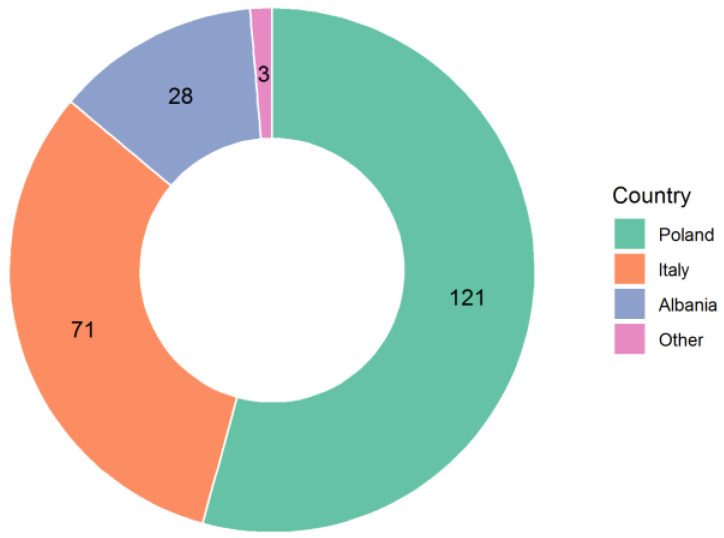
Respondents from different European countries.

**Figure 2:**
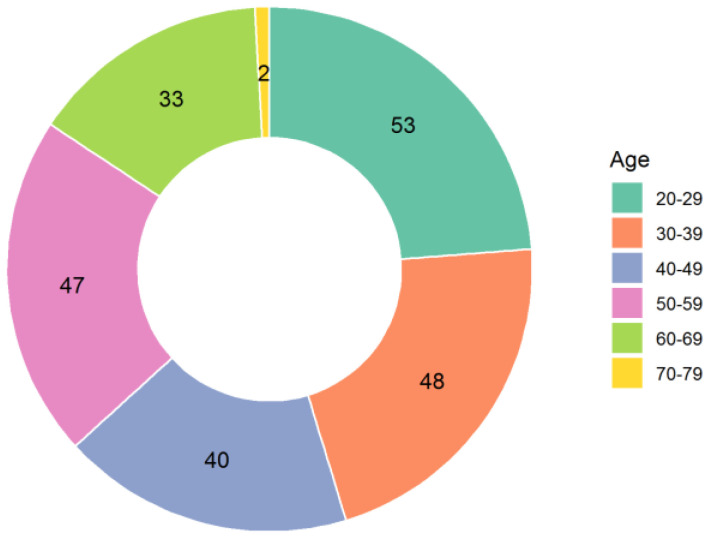
Age ranges of the respondents.

A total of 223 people answered the questionnaire, 57 (25.6%) males and 166 (74.4%) females. The Pfizer/BioNTech vaccine was used in 217 cases, AstraZeneca in 5 cases, and Moderna in 1 case. A total of 182 subjects received both doses, while 41 only received a first dose. The distribution of countries and age groups is shown in Figure 1 and Figure 2.

The age of subjects was considered as a semi-continuous variable with values in the middle of the selected interval. The duration of symptoms was taken in days, with the “1 week” option in the questionnaire being interpreted as 7 days. 

The distribution of binary variables is shown in Table 3.

### 3.2. Correlations

Correlation coefficients between age, gender and symptoms-related characteristics are shown in Table 4.

Between symptoms-related characteristics, the most correlated with age and gender group is “Q13: Time between inoculation and general symptoms onset” and “Q15: Duration of general symptoms presence”. The correlation of gender with Q13 is positive and with Q15—negative, so, on average, males had symptoms later and shorter. Additionally, correlation of age with Q13 is positive and with Q15—negative, so, on average, elder people had symptoms later and shorter. 

### 3.3. Probability of Occurrence of Oral Symptoms 

No factors were discovered to have a significant influence on the occurrence of oral symptoms after the first dose. The very small number of occurrences of such symptoms (7 out of 223, 3.1%) may be partially the reason for this. The model results for the occurrence of oral symptoms after the second dose are shown in Table 5.

Age and the presence of general disease have a significant influence on the probability of the occurrence of oral symptoms. The log of the odds ratio increased by 2.09 in the case of the presence of general disease and decreased by 0.97 for every 10 years of age.

### 3.4. Probability of Occurrence of Facial Symptoms

The model results for facial symptoms occurrence after first and second doses are shown in Table 6 and Table 7, respectively.

General diseases presence has significant influence on the probability of facial symptoms occurrence after first dose, increasing the log of the odds ratio by 2.02.

Autoimmune pathologies presence and age have significant influence on the probability of facial symptoms occurrence after second dose. The log of the odds ratio increases by 1.58 in the case of autoimmune pathologies presence and decreases by 0.91 for every 10 years of age.

### 3.5. Probability of Taking an Absence Day

No factors were found to have a significant influence on taking an absence day after the first dose. The model results for taking an absence day after the second dose are shown in Table 8.

Gender, smoking and regular medication intake have a significant influence on the probability of taking an absence day. The log of the odds ratio increased by 1.14 for smokers, decreased by 1.05 in the case of regular medication intake, and was 0.68 smaller for males than females.

### 3.6. Duration of Symptoms

Factors influencing the duration of symptoms were examined using a linear regression model. The model results for taking an absence day after the first and second doses are shown in Table 9 and Table 10.

Gender and regular medication intake had a significant influence on the duration of symptoms after the first dose. The expected duration of symptoms was 0.62 of a day shorter for males than females and 0.63 of a day shorter in the case of regular medication taking.

Gender, age, and smoking had a significant influence on the duration of symptoms after the second dose. The expected duration of symptoms was 0.33 of a day shorter for males than females, 0.51 of a day longer for smokers, and 0.41 of a day longer with for those with general disease.

## 4. Discussion

The current survey-based study aimed to assess facial and oral manifestations after receipt of a COVID-19 vaccine. The study results showed very few occurrences of such symptoms (7 out of 223, 3.1%, after the first dose; and 9 out of 166, 5.4%, after the second dose) and the vast majority of respondents (97.3%) received the Pfizer/BioNTech vaccine. The study participants were mostly female (74.43%), with 25.56% males. Eight persons reported having diabetes type 1, 30 were smokers, 53 had allergies, 47 reported general disease, and 75 had a regular medication intake. The survey was conducted in the first 2 weeks of March 2021 to allow for the enrolment of subjects with a median follow-up time of 2 months after the second dose. 

The results presented in this study are preliminary. The study will continue over the next 10 months to register more health professionals from other European countries who were contacted through academic contacts but were unable to participate as their countries’ vaccination strategies first targeted the most vulnerable population groups and then medical personnel.

As with adverse drug reactions that may affect the oral cavity, even with vaccination, both the pharmacological mechanism of action of the vaccine and the possibility of oral and facial symptoms should be assessed [9]. As described previously by Tarakji et al., who discussed vaccination against the hepatitis B virus (HBV), most of the oral complications are self-limiting [9]. Furthermore, oral side effects following the administration of other vaccines (i.e., polio or diphtheria), are reported by Riad [8] to be very rare. Research studies about the possible oral manifestations of all types of vaccinations represent only a small portion of total vaccine research and are mostly in the form of case reports [10,11]. The presence of oral mucosal symptoms concomitant with other general dermatological manifestations has been more widely discussed [8,9,12]. For example, oral symptoms after HBV vaccination have been correlated with lichen planus (LP). The association of LP with cases of HBV vaccination is a rare event, which can occur irrespective of the type of vaccine provided for the patient, and manifestation ranges from days to 3 months after any of the three doses of the recommended HBV vaccine [9,12].

The COVID-19 vaccination process might be one of the first seen in the 21st century that has spread worldwide in a short time interval, and on a scale that involves almost all ethnic, geographical and age groups. Such a scenario presents a greater possibility of observing the incidence of oral manifestations, which with the normal seasonal vaccinations might have gone unseen or considered as coincidence [12].

As reported from the Phase 3 clinical controlled trial of the mRNA-based vaccine for COVID-19, the most frequent adverse events, (unsolicited, severe, and serious) were generally similar among participants in the placebo and control groups [13]. This confirms the idea of self-limitation of the symptoms [9]. 

In the current survey, almost all of the participants (97.3%) received the Pfizer-BioNtech vaccine. BNT162b2 is a lipid nanoparticle-formulated, nucleoside-modified RNA vaccine that encodes a prefusion stabilized, membrane-anchored SARS-CoV-2 full-length spike protein. The ongoing efficacy trial showed that a two-dose regimen of BNT162b2 presented a 95% protection against COVID-19 in a population older than 16 years [5].

The list of OAEs reported in the information to patients and healthcare providers in EU are temporary unilateral facial drooping and acute peripheral facial paralysis, respectively. These OAEs are considered rare (up to 1 in 1000 people), and none of the participants in this study reported these adverse events, although this could be explained by the limited sample size. 

The survey questions about facial symptoms investigated changes in sensitivity, facial paresis, facial aesthetics and paralysis. The queries about oral symptoms investigated burning sensations, oral aphthous-like lesions, taste alteration, xerostomia, tongue depapillation, pain, stomatitis/mucositis, commissural cheilitis and oral candidiasis. Changes in sensitivity were reported by 2.7% and 3.1% of the sample after the first and second dose, respectively. Oral symptoms were reported by 3.1% and 4% of the respondents after the first and second dose, respectively. Among the oral manifestations, burning sensation was the most frequent OAE after both the first and second doses, at 2.7% and 3.4%, respectively. Oral aphthous-like lesions were reported by 1.6% and 2.7% after the first and second dose, respectively. Taste alterations were more frequent after the second dose (3.4%) than after the first (1.1%).

The current study evaluated the duration of symptoms generally and did not separately assess the duration of oral and facial symptoms; however, the low incidence of such symptoms justified the survey strategy. Furthermore, in the current study, general diseases along with the age progression had a significant influence on the probability of occurrence of oral symptoms, which is in accordance with previous studies [14]. 

Moreover, the questionnaire investigated possible taste alterations after COVID-19 vaccination, an issue raised before with regard to influenza vaccination [14]. Only three patients out of 223 reported taste alteration. However, the questionnaire was not led by chemosensory testing to confirm whether these factors were directly related to the vaccination, general disease, drug intake, presence/absence of allergies, diabetes type 1 and autoimmune pathologies, or other factors such as the severity of the general adverse reactions after vaccination. 

The struggle to provide the COVID-19 vaccine is significant in most countries. For this reason, the World Health Organization and other key stakeholders in supporting the Coalition for Epidemic Preparedness Innovations (COVAX) support low and middle-income countries by seeking to provide vaccination by December 2021 [15]. In addition, vaccination policies vary among the countries, based on governmental decisions and the needs of the population [16]. At the time of this survey in Poland, the group receiving the vaccination were the so-called “group zero”, which involved medical personnel who agreed to the vaccination. The situation was the same in Italy, and so for such cases, medical personnel from these countries were contacted for the purpose of this study. 

To our knowledge, this is the first report presenting the results of a questionnaire survey within such a short time of the vaccination. The NCBI reports one clinical trial in the same field titled “Oral Side Effects of COVID-19 Vaccine” (clinical trial no NCT04706156), which was conducted in four different European universities in the same geographical region, although the results of this clinical trial will only be published 1 year after the study has begun [17,18,19]. For this reason, no specific results have yet been published from this study. 

### Limitations

The main limitations of this study are (i) the small sample size; (ii) the short time of follow-up; and (iii) the respondent groups not being matched for gender (50:50) and age. However, the research is continuing in order to address these limitations and provide a complete epidemiological picture, with groups defined by sex and age and specific pathologies. The putative date by which it is expected that the final and conclusive report with the appropriate number of patients can be published is the end of 2021.

## 5. Conclusions

Based on the results of this preliminary survey, there is no observed significant correlation between vaccine administration for COVID-19 and facial and oral manifestations. 

## Figures and Tables

**Table 1:** Characteristics of the respondents.

Questionnaire Part A: Characteristics of Respondents		
Q1: Country	Italy Poland France Germany Switzerland Albania Denmark Norway United Kingdom Spain Other	71 (31.9%) 121 (54.3%) 28 (12.6%) 1 (0.4%) 1 (0.4%) 1 (0.4%)
Q2: City	…….	
Q3: Gender	Female Male	166 (74.4%) 57 (25.6%)
Q4: Age	20–29 30–39 40–49 50–59 60–69 70–79	53 (23.8%) 47 (21.5%) 41 (17.9%) 47 (21.1%) 33 (14.8%) 2 (0.9%)
Q5: Profession	Student Medical Doctor Dentist Nurse Other	13 (5.9%) 35 (15.3%) 69 (30.5%) 76 (34.7%) 30 (10.9%)
**Questionnaire Part D—Underlying medical conditions and lifestyle factors**		
Q22: Are you a smoker?	Yes No	30 (13.5%) 193 (76.7%)
Q23: Are you an allergic subject?	Yes No	53 (23.8%) 170 (76.2%)
Q24: What are you allergic to?	…….	
Q25: Do you have any general diseases?	Yes No	47 (21.1%) 176 (78.9%)
Q26: Please list if any general diseases you have.	…….	
Q27: Do you have any autoimmune disease?	Yes No	18 (7.6%) 205 (92.4%)
Q28: Do you have diabetes type 1?	Yes No	8 (3.6%) 215 (96.4%)
Q29: How would you describe your diet?	Omnivorous Vegetarian Flexitarian Vegan	210 (94.2%) 11 (5) 2 (0.8%)
Q30: Do you have rapid mood swings?	Yes No	43 (19.3%) 180 (80.7%)
Q31: Do you take any regular medication?	Yes No	75 (33.6%) 148 (66.4%)
Q32: If you do take any regular medication, please list it here.	…….	

**Table 2:** The answers given by the respondents.

Questionnaire Part B: COVID-19 Vaccine, Number of Doses, Date of Inoculation, Premedication, General Symptoms.			
		First or single dose	Second dose
Q6: Vaccine dose	First one Second one Single dose vaccine	41 (18.4%)	182 (81.6%)
Q7: Type / name of COVID-19 vaccine	Pfizer/BioNTech Moderna Astrazeneca Sputnik Other	35 (15.7%) 1 (0.4%) 5 (2.3%)	182 (81.6%)
Q8: Vaccination date (first dose)	…….	…….	…….
Q9: Vaccination date (second dose)	…….	…….	…….
Q10: Vaccination date (single dose)	…….	…….	…….
Q11: In anticipation of the vaccine, did you take any medicine?	No Antipyretics Analgesics Anti-inflammatory	203 (91.1%) 7 (3.1%) 9 (4%) 4 (1.8%)	114 (62.6%) 35 (19.2%) 21 (11.5%) 12 (6.7%)
Q12: If you took any medicine in anticipation of the vaccine, can you describe the medication and the total dose.	…….	…….	…….
Q13: What was the time between inoculation and onset of general symptoms?	No symptoms 1 h 2 h 3 h 6 h 12 h 24 h	103 (45.9%) 11 (4.6%) 10 (4.6%) 14 (6.2%) 33 (15%) 34 (15.5%) 18 (8.2%)	49 (13.9%) 8 (4.7%) 2 (1.2%) 18 (10.5%) 32 (18.6%) 50 (29%) 23 (22.1%)
Q14: Did your symptoms include fever?	No Yes, from 36.6 to 37.4 degree C Yes, from 37.5 to 38.0 degree C Yes, from 38.1 to 39.0 degree C	185 (82.8%) 25 (11.2%) 8 (3.7%) 5 (2.3%)	101 (54.7%) 47 (26.3%) 22 (12.3%) 12 (6.7%)
Q15: How long were the general symptoms present?	No symptoms 1 day 2 days 3 days 4 days 1 week	137 (61.5%) 43 (19.3%) 23 (10.3%) 11 (4.9%) 6 (1.7%) 3 (1.3%)	50 (27.5%) 62 (34.1%) 48 (26.4%) 18 (9.9%) 1 (0.5%) 3 (1.6%)
Q16: Did you experience loss of concentration?	Yes No	26 (11.6%) 197 (88.4%)	57 (32.4%) 125 (67.6%)
Q17: Did you need to take an absence day?	Yes No	8 (3.6%) 215 (93.3%)	53 (23.7%) 129(57.4%)
**Questionnaire Part C—Facial and Oral Manifestations**			
Q18: Facial symptoms (changes in sensitivity, facial paresis, facial aesthetic paralysis)	Yes No	6 (2.7%) 217 (97.3%)	8 (4.2%) 174 (95.8%)
Q19: Oral symptoms	Yes No	7 (3.1%) 216 (95.5%)	9 (4%) 173 (76.6%)
Q20: Oral manifestations	No oral manifestations Burning sensation Oral aphthous-like lesions Taste alteration Xerostomia Tongue depapillation Pain Stomatitis/mucositis Commissural cheilitis Oral candidiasis	216 (97%) 3 (1.3%) 3 (1.3%) 1 (0.4%)	161 (88.7%) 5 (2.7%) 1 (0.5%) 4 (2.2%) 5 (2.7%) 1 (0.5%) 5 (2.7%)
Q21: If you experienced other oral manifestations, please list them below.	…….	…….	…….

**Table 3:** Distribution of binary variables.

Variable	Yes	No	No Answer
Oral symptoms (dose 1)	7	213	3
Oral symptoms (dose 2)	9	171	2
Absence day (dose 1)	8	208	7
Absence day (dose 2)	53	128	1
Diabetes	8	215	–
Smoker	30	171	22
Allergic	53	170	–
Regular medication	75	148	–
General diseases	47	176	–

**Table 4:** Correlation coefficients. Significant values are in bold, *p*-values in parentheses. A positive value in the age column means that older people had higher expected values of the correspondent symptoms-related characteristic. A positive value in the gender column means that males had higher expected values of the correspondent symptoms-related characteristics.

Symptoms-Related Characteristic	Age	Gender
Time between inoculation and general symptoms onset (first dose)	**0.135** (0.064)	**0.204** (0.005)
Time between inoculation and general symptoms onset (second dose)	**0.223** (0.003)	**0.232** (0.002)
Fever (first dose)	–0.079 (0.285)	–0.109 (0.138)
Fever (second dose)	**–0.298** (<0.001)	–0.047 (0.537)
Symptoms Duration (first dose)	**–0.152** (0.035)	**–0.215** (0.003)
Symptoms Duration (second dose)	**–0.207** (0.006)	**–0.128** (0.094)
Concentration Loss (first dose)	–0.108 (0.114)	–0.021 (0.763)
Concentration Loss (second dose)	**–0.333** (<0.001)	–0.001 (0.994)
Absence Day (first dose)	–0.077 (0.258)	**–0.116** (0.089)
Absence Day (second dose)	**–0.129** (0.083)	–0.099 (0.185)
Facial Symptoms (first dose)	–0.010 (0.896)	–0.028 (0.709)
Facial Symptoms (second dose)	**–0.148** (0.079)	0.017 (0.838)
Oral Symptoms (first dose)	0.059 (0.386)	–0.107 (0.113)
Oral Symptoms (second dose)	**–0.175** (0.019)	–0.028 (0.705)

**Table 5:** Logistic model results for occurrence of oral symptoms after the second dose.

Oral Symptoms, Second Dose				
Variable	Estimate	Std. Error	z–Value	*p*-Value
(Intercept)	–0.111	1.181	–0.094	0.925
Age	–0.097	0.038	–2.573	0.010
General disease	2.090	0.783	2.669	0.007
Model characteristics	AIC: 64.31 McFadden’s R^2^ = 0.184			

**Table 6:** Logistic model results for facial symptoms occurrence after first dose.

Facial Symptoms, First Dose				
Variable	Estimate	Std. Error	z-Value	*p*-Value
(Intercept)	–3.839	0.682	–5.631	<0.001
Smoker	1.424	0.979	1.455	0.146
RegularMedication	–2.014	1.401	–1.437	0.151
GeneralDiseases	2.021	1.167	1.733	0.083
Model characteristics	AIC: 47.154 McFadden’s R^2^ = 0.142			

**Table 7:** Logistic model results for facial symptoms occurrence after second dose.

Facial Symptoms, Second Dose				
Variable	Estimate	Std. Error	z–Value	*p*-Value
(Intercept)	–0.363	1.539	–0.236	0.814
Age	–0.091	0.048	–1.894	0.059
Autoimmune Pathologies	1.577	0.507	3.112	0.002
Model characteristics	AIC: 42.165 McFadden’s R^2^ = 0.273			

**Table 8:** Logistic model results for taking an absence day after second dose.

Taking an Absence Day, Second Dose				
Variable	Estimate	Std. Error	z-Value	*p*-Value
(Intercept)	–0.586	0.227	–2.584	0.010
Gender—male	–0.676	0.408	–1.655	0.098
Smoker	1.135	0.469	2.417	0.016
Regular medication	–1.048	0.404	–2.597	0.009
Model characteristics	AIC: 211.52 McFadden’s R^2^ = 0.070			

**Table 9:** Linear model results for duration of symptoms (days) after first dose.

Duration of Symptoms, First Dose				
Variable	Estimate	Std. Error	t-Value	*p*-Value
(Intercept)	1.4201	0.1413	10.048	<0.001
Gender—male	–0.618	0.237	–2.602	0.010
Regular medication	–0.627	0.222	–2.820	0.005
Model characteristics	R^2^ = 0.076 F[2189] = 7.75; *p* < 0.001			

**Table 10:** Linear model results for duration of symptoms (days) after second dose.

Duration of Symptoms, First Dose				
Variable	Estimate	Std. Error	t-Value	*p*-Value
(Intercept)	1.982	0.283	7.013	<0.001
Gender—male	–0.332	0.200	–1.664	0.098
Age	–0.0166	0.0065	–2.552	0.012
Smoker	0.509	0.267	1.909	0.058
General disease	0.405	0.234	1.729	0.086
Model characteristics	R^2^ = 0.076 F[4169] = 3.464; *p* = 0.009			

## Data Availability

Data are available upon request.
